# Evaluation of fatty acid‐related nutritional quality indices in fried and raw nile tilapia, (*Oreochromis Niloticus*), fish muscles

**DOI:** 10.1002/fsn3.1760

**Published:** 2020-08-02

**Authors:** Mengistu F. Mekonnen, Derese T. Desta, Fikadu R. Alemayehu, Gezahegn N. Kelikay, Alemneh K. Daba

**Affiliations:** ^1^ School of Nutrition, Food Science and Technology College of Agriculture Hawassa University Hawassa Ethiopia; ^2^ College of Medicine and Health Science Hawassa University Hawassa Ethiopia

**Keywords:** Ethiopia, fatty acids, fried fish muscle, Lake Hawassa, nutritional quality index

## Abstract

During frying, fat degrade and many reactions occur resulting in numerous altered fatty acid products. This would lead to the formation of *Tran's* fatty acids and changes in nutritional qualities. Hence, this study was aimed to determine the changes during frying on fatty acid composition of Nile tilapia (*Oreochromis Niloticus*) fish muscle from local fish market of Hawassa, Ethiopia. Fish fryers reported that they usually use palm oil for frying. They also indicated that the oil is kept for days and reused for frying at different cycle. In fried fish, 35 types of fatty acids were identified and 27 in raw fish muscle. Palmitic, stearic, heptadecanoic, and tetradecenoic acids were the abundant saturated fatty acids in both fried and raw fish muscle. Oleic, Docosahexaenoic, Eicosapentaenoic, and Linoleic acid were the major unsaturated fatty acids. The percentages of total saturated fatty acids (SFA) of raw fish muscle (47.4%) were found to be lower compared with fried fish muscle (51.8%). The *n*‐6*/n*‐3 ratio (7.83), index of atherogenicity (0.85), and thrombogenicity (1.71) in the fried fish muscle were in the undesirable values. The hypocholesterolemic/hypercholesterolemic ratio was relatively similar in the fried (1.09) and raw (1.02) fish muscles. The total unsaturated fatty acids (UFA) level of raw fish muscle (52.2%) was higher than the fried fish muscle (47.2%). Higher percentage of *Tran's* fatty acid was identified in the fried fish muscle compared with raw. Frying considerably altered fatty acid composition of fish muscle. It increased saturated fatty acid concentration and decreased unsaturated. Hence, frying noticeably decreases the nutritional quality of fish muscle. Therefore, it is suggested that further analysis on fatty acid composition of oil usually used for frying and the nutritional quality index across frying cycles.

## Graphical abstract

Frying fish muscle considerably alters fatty acid compositions by increasing saturated fatty acids and decreasing unsaturated fatty acids instead. Hence, frying considerably decreases the nutritional quality of fish muscle.

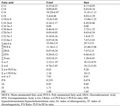

## 1 INTRODUCTION

Fish is a very nutritious aquatic animal that can provide a range of micronutrients, complete amino acid and fatty acid in a small amount (FAO, 2008). Interest in fish consumption increased due to the high content of health significant omega‐3 PUFAs, particularly eicosapentaenoic acid (20:5n‐3, EPA) and docosahexaenoic acid (22:6n‐3, DHA). The long chain n‐3 polyunsaturated fatty acids, including α‐linolenic acid (ALA), eicosapentaenoic acid (EPA), and docosahexaenoic acid (DHA), are important to human health, as they have been reported to protect against cardiovascular disease and neurodegeneration and to reduce inflammation(Leung, Galano, Durand, & Lee, 2018). Besides playing important role in cardiovascular and inflammatory diseases, the essential fatty acids are vital in the development of neuron in infants and in fat glycolic control. Consumption of fish reduces morbidity and mortality from coronary heart diseases, and among women, it reduces risks of suboptimal neurodevelopments of their offsprings. In fact, fish‐based food products are not risk free at all. They require safety and quality‐related emphasis across fish food chains. The risks could be linked with production, processing, and consumption of fish and fish products (FAO, 2014). Frying is one of the complex processes which could modify nutrient composition of the fried food product (Sun et al., 2019).

It is evident frying of foods has gained more and more acceptance all over the world although some physiological drawbacks have been recognized for degraded frying fats and fried foods for many years. Nevertheless, the fried products are estimated for their crispy texture, the roasted, fried aroma, and their pleasant golden to brown color (Brühl, 2014). However, it is known from earlier study that the type of preparation has some influence on the final fatty acid (FA) composition of the product. Especially during frying, the fat used will have some influence on the final product (Sampels, Zajíc, & Mráz, 2014). For instance, during frying oils and fats degrade and many reactions occur resulting in numerous altered fatty acid products. The geometrical isomerization of double bonds leads to the formation of *Trans* fatty acids (Brühl, 2014). Consumption of formed *Trans* and saturated fat is considered as one of the principal factors increasing risks of heart disease, cancer, diabetes, and hypertension. To reduce these health impacts, FAO (2014) recommends that countries need to have risk inspection system. However, controlling system which could reduce arising health impacts is not well established in the developing countries including Ethiopia. Findings to support the strengthening process are also limited.

Lake Hawassa is one of important lakes in Ethiopia that contributes for tourism industry and fishing and related income generation activities. Many individuals are dependent on the fish produced in open market (Yirgu, 2011). The most abundant fish (Nile tilapia) is being consumed and sold in existing open fish markets. The type of fish food mainly served in the fish market includes fish fillets (Raw fish muscle) and fried fish. Though frying is claimed for its negative health effect, evidences about effects of frying practices by the fryers in the Hawassa open fish market are very limited. Therefore, this study aims to determine fatty acid profile changes and nutritional quality indices due to frying compared with the raw fish fillets. It is believed to provide evidences for further risk management.

## 2 MATERIALS AND METHODS

### 2.1 Description of the sample collection area

The sample was collected from Hawassa fish market. Hawassa is the capital city of Southern Nations Nationalities and Regional State (SNNPR), which is 273 km south from Addis Ababa. Lake Hawassa is the natural beauty of Hawassa city. The lake is 95.8 square kilometers wide. It stretches 16 km from north east to south west direction and 8 km from northeast to southeast direction, and it has estimated volume 1.036 × 109m^3^ (Kebtieneh, Alemu, & Tesfa, 2016) with maximum depth of 21.6 m. It is found in 6°33′–7°33′ N and 38°22′–38°29′ E. The lake receives surface inflow mainly containing factories and domestic waste through *Tikur Wuha* River. The lake is located in a closed basin and has no surface outflow. Its local name is called “*yefikir hayk*‐in Amharic meaning adorable lake,” and it is very useful mostly for recreation together with production of fish species (Gebremedhin & Berhanu, 2015). The most abundant and important commercial species of fish from Lake Hawassa is Tilapia (*Oreochromis Niloticus*), and its local name called “*koroso*” (Kebtieneh, Alemu, & Tesfa, 2016). It is being consumed and sold in existing open fish markets. The type of fish food mainly served in the fish market includes fish fillets (raw fish muscle) and fried fish, commonly Nile tilapia fish species (Lema Abelti, 2017).

### 2.2 Sample collection and preparations

Fish samples were collected from Lake Hawassa open fish market, Ethiopia. Prior to sample collection, interviews were conducted in order to assess the existing fish frying practices among 10 randomly selected fryers from Hawassa open market were included. A total of 18 unions involved in fish frying business were identified where the samples are selected. The selected open markets were Amora Gedel, Fikir Hayk, and Tikur Wuha. Following the interviews, commonly consumed fresh fish, Nile tilapia (*Oreochromis niloticus*), was purchased from local fishermen of Hawassa fish market mainly at Amora Gedel and Tikur Wuha. A total of representative fried and raw fish Nile tilapia *(Oreochromis* *niloticus)* samples (*n* = 21 each) were included in the study. The samples were categorized based on their sizes from the previous study. The categories were large (>27 cm), medium (18‐27 cm) and small (<19 cm) for both catchments (Alemu, 2017). During the sample collection, history showing the sources of fish purchased was taken at each site. Freshly collected adult fish (fried and raw) individuals were thawed and dissected carefully to obtain muscle tissue. The separated tissues were frozen in ice box until keep at −20°C in deep freezer unit. The frozen samples were transported to JIJE Analytical *Testing* Service Laboratory, Addis Ababa, Ethiopia. This analytical testing service laboratory was ISO 17025:2005 accredited for Agricultural and Nutrition samples by Ethiopian National Accreditation Office (ENAO). Composite fish muscle tissues for fatty acid analysis were taken from each specimen. Samples were homogenized by passing them twice through a mincer with 4 mm holes and mixed thoroughly. The resultant homogenate was packed into several small convenient sterile containers and stored at 0°C prior to laboratory analysis(Gladyshev, Sushchik, Gubanenko, Demirchieva, & Kalachova, 2007).

### 2.3 Fat (lipids) extraction and preparation of fatty acid methyl esters (FAMEs)

Fat was extracted by using Soxhlet method as per the AOAC 1998. The extraction flasks were cleaned, dried in drying oven (Memmert) at 105°C for 1 hr, cooled in desiccators (with granular silica gel) for 30 min, and then weighed. Homogenized sample was weighed into a conical flask and dried for 1 hr at 105°C. The flask was cooled to room temperature, and 50 ml of 4 M hydrochloric acid was added. The solution was boiled for 1 hr. Then, 150 ml of water was added, and the solution was filtered through fluted filter paper and washed until neutral reaction on litmus paper. The filter paper was dried with its content for 1 hr at 105°C and inserted in extraction thimble of Soxhlet apparatus. The lipids were extracted with petrol ether or hexane into weighted round bottom flask for 4 hr on the sand bath other heating apparatuses. After extraction, the light petrol ether or hexane evaporated and the flask was dried at 105°C and weighed for calculating the total fat content of the sample. Fatty acid profile was determined by gas chromatography mass spectrophotometer (GCMS) (Petrović, Kezić, & Bolanča, 2010).

The fatty acid methyl esters (FAME) of the fish fats were then analyzed by capillary gas chromatography. The initial temperature of the column was set at 160°C and finally increased to 240°C at a rate of 3°C/min. The detector temperature was set at 270°C, while the temperature at the injection port was maintained at 240°C. Helium was used as the carrier gas at 14 psi. Relative quantities were expressed as weight percent of total fatty acids identified via comparison of retention times to known FAME standards (Petrović et al., 2010).

### 2.4 Nutritional lipid quality indices

Nutritional indicators determining the nutritional quality of both fried and raw fish muscles were calculated. Index of *n*‐6/*n*‐3 ratio refers to the comparison of the *n*‐6 PUFAs over *n*‐3 PUFAs. Index of P/S ratio refers to the fraction of the PUFAs over SFAs. Indices of atherogenicity (IA), thrombogenicity (IT), and hypocholesterolemic/hypercholesterolemic fatty acids (HH) were calculated with following formulas (Estuary et al., 2020; Palacin‐arce, Monteagudo, Beas‐jimenez, Olea‐Serrano, & Mariscal‐Arcas, 2015).

$$\text{IA}\;=\;\left[\left(C12:0\;+\;\left(4\;\times \;C14:0\right)\;+\;C16:0\right)\right]/\left(\text{MUFAs}\;+\;n‐6\;\text{PUFAs}\;+\;n‐3\;\text{PUFAs}\right)\;$$

$$\text{IT}\;=\;\left(\text{C14}:0\;+\;\text{C16}:0\;+\;\text{C18}:0\right)/\left[\left(0.5\;\times \;\text{MUFAs}\right)\;+\;\left(0.5\;\times \;n‐\text{6 PUFAs}\right)\;+\;\left(3\;\times \;n‐\text{3 PUFAs}\right)\;+\;\left(n‐3\;\text{PUFAs}/n‐\text{6 PUFAs}\right)\right]$$

$$\text{HH}\;=\left(\text{C18}:1n‐9\;+\;\text{C18}:2n‐6\;+\;\text{C2}0:4n‐6\;+\;\text{C18}:3n‐3\;+\;\text{C2}0:5n‐3\;+\;\text{C22}:5n‐3\;+\;\text{C22}:6n‐3\right)/\left(\text{C14}:0\;+\;\text{C16}:0\right)\;$$

### 2.5 Statistical analysis

Upon the completion of laboratory analysis, all individual fatty acids were identified. The identified fatty acids were categorized under unsaturated (UFA) and saturated fatty acids (STA). Each category was reported by percentages and standard deviations. The *n*‐6*/n*‐3 ratio, IA, IT, and HH were calculated to evaluate the nutritional quality both in fried and raw fish muscles. One‐way analysis of variance (ANOVA) with SPSS v.20 with Tukey's test at a 5% significance level (*p < *.05) also employed to compare the variation in fatty acid with respect to different size, fried and raw fish muscles.

## 3 RESULTS

### 3.1 Fish frying practices among the fish fryers of Hawassa open fish market

Preliminary background information on fish frying practice was collected in market from where the fried fish samples taken. Accordingly, the respondents reported that most abundant and preferred and abundant species for frying was Nile tilapia (*Oreochromis niloticus*), and they locally call it “*Koroso*.” The fryers purchase from unions and usually keep it a refrigerator until frying. The fish fryers use mainly a metal pan and firewood. They reported that on average, they fry five fishes at a time using a single pan in open air. It takes them on average 15–20 min to fry fish in a single cycle. The fryers reported that mostly they use palm oil as a type of oil for frying the fish. They reuse the oil until it becomes very black, an indication to stop using it more. They reported that the used oil is even kept for days and reused again for frying. The fryers perceive that reusing oil is advantageous in many ways. They think that the reused oil can improve the flavor and taste characteristics of the fried fish, and hence, the customers usually prefer that. The fryers were asked whether they have been informed about the food safety issues of frying fish. They responded as they know as fish is easily spoiled product and regarded as they are not well informed. They never had been gone for medical checkups in general.

### 3.2 Dominant fatty acids of Nile tilapia, (*oreochromis niloticus*), fish muscles

A total of 35 and 27 fatty acids were identified in the fried and raw Nile tilapia fish muscles, respectively. Palmitic acid, stearic acid, heptadecanoic acid, and tetradecenoic acid were the abundant saturated fatty acids in both fish muscles. Oleic acid, docosahexaenoic acid, eicosapentaenoic acid, and linoleic acid were also the major unsaturated fatty acids. Furthermore, the raw fish muscle contained considerable amounts of higher percentages of unsaturated fatty acids. The dominant fatty acids are listed below in Table 1.

**Table 1:** Dominant fatty acids identified both in raw and fried Nile tilapia (*Oreochromis niloticus*) fish muscle

Common name	Chemical formula	Level of saturation	Raw	Fried
4,7,10,13,16‐Docosapentaenoate	C23H36O2	Unsaturated	√	—
9,10‐Octadecenoic acid	C19H38O4	Unsaturated	—	√
Arachidonic acid	C21H34O2	Unsaturated	√	—
cis‐5,8,11,14,17‐Eicosapentaenoic acid	C21H32O2	Unsaturated	√	—
Cis‐8,11,14‐Eicosatreinoic acid	C21H36O2	Unsaturated	√	—
Docosahexaenoic acid	C23H34O2	Unsaturated	√	√
Docosapentaenoic acid	C21H32O2	Unsaturated	√	—
Elaidic acid	C19H36O2	Unsaturated	√	—
Linoleic acid	C19H34O2	Unsaturated	√	√
Oleic acid	C19H36O2	Unsaturated	√	√
Palmitoleic acid	C17H32O2	Unsaturated	√	√
Heptadecanoic acid	C18H36O2	Saturated	√	—
Palmitic acid	C16H32O2	Saturated	√	√
Stearic acid	C19H38O2	Saturated	√	√
Tetradecenoic acid	C15H30O2	Saturated	√	√

### 3.3 Nutritional quality index (NQI) of fried and raw fish muscles

Figures of each value of fatty acids for fried and raw fish muscles are depicted below in the Table 2. The average percentages of C12 and C14 are lower in the raw fish muscle compared with fried fish muscle. Surprisingly, the figures for C16 and C18 were found to be higher in the raw fish muscle. Out of total polyunsaturated fatty acids, percentages of docosahexaenoic acid (DHA) and eicosapentaenoic acid (EPA) were (7.67%), (0.65%), and (0.87), (0.20), respectively, for raw and fried fish muscles. The ratio of polyunsaturated fatty acid and saturated fatty acid in raw fish muscle was 0.47, and this is decreased to 0.22 in fried fish muscles. The ratio of Omega 3 and Omega 6 fatty acid (ω‐3/ω‐6) was equivalent to 1 in raw fish sample, and this was 0.24 in the fried fish muscle. Both the nutritional quality indices are reduced. The level of *Trans* fatty acid percentages out of polyunsaturated fatty acid accounted (1.28%) in raw and (4.02%) in fried fish muscle, respectively. On the other hand, it is known that the existing fatty acids in the fish muscles contribute for the human health. Therefore, it is important to evaluate the health contribution using different indices. These indices related to these could be such as, *n*‐6/*n*‐3 ratio, P/S ratio, index of atherogenicity (IA), index of thrombogenicity (IT), and hypocholesterolemic/hypercholesterolemic index (HH). In the current study, the ratio of *n*‐6/*n*‐3 was 7.83 for fried fish muscle and 0.90 for raw fish muscle. Hence, the ratio is considerably increased due to frying. The ratio of polyunsaturated fatty acids to saturated fatty acid (P/S) was 0.216 in the fried which is lower than the raw fish muscle (0.474). The atherogenicity and thrombogenicity index were 0.85 and 1.71 in fried and 0.68 and 0.78 in raw fish muscle, respectively. The ratios are relatively lower in the fried fish muscle. The hypocholesterolemic/hypercholesterolemic index was 1.09 in fried and 1.02 in raw fish muscle.

**Table 2:** Comparison of fatty acid composition(expressed as percentage of total fatty acids) and nutritional quality indices (NQI) of Nile tilapia (*Oreochromis niloticus*) fish muscle

Fatty acid	Fried	Raw
C12	0.25 ± 0.22	0.17 ± 0.05
C14	0.09 ± 0.01	0.43 ± 0.16
C16	38.29 ± 6.87	31.41 ± 1.13
C18	5.4 ± 0.48	8.03 ± 2.29
C181n‐9	32.0 ± 5.05	13.08 ± 1.25
C18 2n‐6	8.16 ± 3.17	6.45 ± 0.46
C18:3n‐3	0.00	0.00
C20:4n‐6	0.46 ± 0.33	2.75 ± 1.25
C20:5n‐3	0.05 ± 0.05	0.65 ± 0.34
C22:5n‐3	0.18 ± 0.16	1.8 ± 0.75
C22:6n‐3	0.87 ± 0.38	7.67 ± 3.83
∑MUFA	35.84 ± 2.9	29.7 ± 3.9
∑PUFA	11.36 ± 1.2	22.48 ± 5.08
∑DHA	0.87 ± 0.24	7.67 ± 2.4
∑EPA	0.20 ± 0.12	0.66 ± 0.21
∑*Trans*	4.01 ± 0.21	1.28 ± 0.41
Σω‐3	2.12 ± 1.19	10.12 ± 4.91
Σω‐6	8.76 ± 3.45	10.12 ± 2.03
Σ*n*‐6 PUFAs	8.62	9.20
Σ*n*‐3 PUFAs	1.10	10.12
*n*‐6/*n*‐3	7.83	0.91
P/S	0.219	0.474
IA	0.85	0.68
IT	1.71	0.78
HH	1.09	1.02

Abbreviations: DHA, docosahexaenoic acid; EPA, eicosapentaenoic acid; HH, hypocholesterolemic/hypercholesterolemic ratio; IA, index of atherogenicity; IT, index of thrombogenicity; MUFA, monounsaturated fatty acid; *n*‐6*/n*‐3 ratio, *n*‐6 PUFAs/*n*‐3 PUFAs ratio; P/S ratio, PUFAs/SFAs ratio; PUFA, polyunsaturated fatty acid.

Besides, as it is shown in Table 3 large fried fish got the highest composition of saturated fatty acids (58.8%) than medium and small size fish. Saturated fatty acids of large size raw fish muscle were found to be 49.51% of the total fatty acids. The percentages of total unsaturated fatty acids of raw and fried large size were found to be 50.4% and 38.35%, respectively. Compared with the other sizes, the medium size showed a slight difference in total unsaturated fatty acid percentages. Unsaturated fatty acid percentages were relatively high in small size raw fish (54.26%) than the others. Therefore, the size of fish is believed to be one of the factors for fatty acid composition variations (Table 4).

**Table 3:** Fatty acid composition (expressed as percentage of total fatty acids) of Nile tilapia, (*Oreochromis niloticus*), across sizes

Sum of fatty acids	Fried			Raw		
	Larger	Medium	Smaller	Larger	Medium	Smaller
∑SFA	55.3	49.9	50.3	48.2	47.4	46.6
∑UFA	42.7	49.6	49.2	51.3	52.1	53.2
∑MUFA	30.5	38.1	38.94	33.57	33.1	22.57
∑PUFA	7.85	13.89	12.34	16.83	18.91	31.69
∑DHA	0.65	1.005	0.97	6.17	7.01	9.8
∑EPA	0.31	0.15	0.13	0.54	0.56	0.87
∑*Trans*	3.81	4.14	4.08	1.3	1.25	1.23

Note

The samples were categorized based on their sizes from the previous study. Larger: >27 cm, medium: 18‐27 cm, and smaller: <19 cm for both catchments (Alemu et al., 2017).

**Table 4:** One‐Way ANOVA output showing effect of size and frying on fatty acid compositions of Nile tilapia (*Oreochromis niloticus*) fish muscle

Fatty acids	Sources	SS	*df*	MS	F	*p*
Saturated fatty acids	Fish size	28.256	2	14.128	2.171	.195
	Type of fish muscle	58.963	1	58.963	9.059	.024*
	Size × type of fish muscle	11.089	2	5.545	0.852	.472
Unsaturated fatty acids	Fish size	42.978	2	21.489	2.054	.209
	Type of fish muscle	75.38	1	75.38	7.205	.036*
	Size × type of fish muscle	19.677	2	9.838	0.94	.441
DHA	Fish size	8.409	2	4.205	1.714	.258
	Type of fish	138.72	1	138.72	56.558	<.0001*
	Size × type of fish muscle	6.576	2	3.288	1.341	.33
EPA	Fish Size	0.042	2	0.021	0.886	.46
	Type of fish muscle	0.642	1	0.642	27.038	.002*
	Size × type of fish muscle	0.137	2	0.069	2.885	.132
*Trans*	Fish size	0.042	2	0.021	0.98	.428
	Type of fish muscle	22.413	1	22.413	1,053.762	<.0001*
	Size × type of fish muscle	0.08	2	0.04	1.874	.233

* Significant at *p* = .05.

The effect of frying fish muscle and the fish sizes on fatty acid composition is shown in Table 4. There was no statistical significant interaction effects of variables between the size of fish and frying on fatty acid compositions (*p* > .05). However, fried fish muscle got significant difference in fatty acid compositions compared with raw (*p* < .05).

## 4 DISCUSSION

The major aim of the present study was to determine the effect of frying fish muscle on fatty acid composition of Nile tilapia fish muscles sampled from Hawassa fish markets. Hence, it was found that a total of 37 fatty acids were identified. The abundant saturated fatty acids were palmitic acid, stearic acid, heptadecanoic acid, and tetradecenoic acid in all fish muscles. Oleic acid, docosahexaenoic acid, eicosapentaenoic acid, and linoleic acid were also the major unsaturated fatty acids. This results are very similar to the other previous study findings conducted in other international and Ethiopia (Lema Abelti, 2017; Robert, Mfilinge, Limbu, & Mwita, 2014). Usually, these fatty acids are considered as the most saturated fatty acids in nature (Oluwaniyi, Dosumu, & Awolola, 2017). As it is shown in the Table 5, the total saturated fatty acid from the raw Nile tilapia fish muscle was found to be 47.4%. Previous study done in Ziway, Ethiopia, showed considerably similar findings (Lema Abelti, 2017). However, this value was found to be higher than the study done in Nigeria (36.56%) (Oluwaniyi et al., 2017). The difference with the earlier study might be due to the natural ecosystem mainly affecting the fish nutrition.

**Table 5:** Comparison of fatty acid composition (expressed as percentage of total fatty acids) of Nile tilapia (*Oreochromis niloticus*) fish muscle

Fatty acids	Fried	Raw	*p*‐value
∑SFA	51.83 ± 3.81	47.4 ± 1.05	.021
∑UFA	47.2 ± 4.8	52.2 ± 1.2	.034
∑MUFA	35.84 ± 2.9	29.7 ± 3.9	.012
∑PUFA	11.36 ± 1.2	22.48 ± 5.08	.001
∑DHA	0.87 ± 0.24	7.67 ± 2.4	<.001
∑EPA	0.20 ± 0.12	0.66 ± 0.21	<.001
∑*Trans*	4.01 ± 0.21	1.28 ± 0.41	<.001
Σω‐3	2.12 ± 1.19	10.12 ± 4.91	.589
Σω‐6	8.76 ± 3.45	10.12 ± 2.03	.099

Note

The *p*‐values for mean and standard deviations were taken from independent sample *t* tests.

The total saturated fatty acids of fried Nile tilapia in the current study were found to be 51.8% of the total fatty acids which were higher compared with other study done in Nigeria (42.4%) (Oluwaniyi et al., 2017). In both studies, the type of oil used was palm oil. However, the difference would possibly be due to the difference in the number of cycle that the same oil used for frying. It was reported also fish fryers in Hawassa uses the same oil for more than eight times in different cycles. This could probably increases the percentages of saturated fatty acids due to the alterations of fatty acids. The mechanism could probably be the exposure of frying oil to higher temperature, atmospheric air, and moistures during the frying stages as well as other occurrences of chemical reactions including oxidations. Other study supports the current finding that different fats and oils can alter the fatty acid composition (Rahimabadi & Dad, 2012; Sampels et al., 2014).

In addition to the frying oil, method of preparation has also effect on fatty acid composition. The changes could be on both saturated an unsaturated fatty acids (Oluwaniyi et al., 2017). Others studies prove still these occurrences. For instance, Beigi, Alizadeh, Rahimabadi, and Elahi (2014) results suggest that frying oil can change the fatty acid composition of fish. Frying oil and method of frying could influence the change. They stated that fatty acids composition responds differently to frying oils. In comparison with raw samples, frying caused to decrease in the content of other fatty acids with exception of linoleic and stearic acids content. In fried samples by canola oil for instance, the increase was happened in the content of oleic acid (C18:1) and linoleic linoleic acid (C18:2). The fried sample by sunflower increased linoleic acid and oleic acid in comparison with raw samples. In raw fish, saturated fatty acid was the most abundant fatty acid (36.810% of total fatty acids) followed by polyunsaturated fatty acids (32.745% of total fatty acids) and monounsaturated fatty acids (30.745% of total fatty acids). This arrangement was changed by frying which was dependent on frying oil and frying method (Beigi et al., 2014).

In the current study, the total unsaturated fatty acid from the raw fish was 52.2% which is less than the study done by the same author in Nigeria (63.4%). Linoleic (C18.2 *n*‐6), EPA (C20.5 *n*‐3), and DHA (C22.6 *n*‐3) present in the raw fish samples. The total unsaturated fatty acid of fried fish muscle was 47.21% which was less the study done in Nigeria (57.4%) (Oluwaniyi et al., 2017). The reason for the difference again probably goes to the cycles and time of frying. In the Nigerian study, the time for frying was 15 minutes and the cycle was one. Whereas the frying time in the current study was more 15 minutes and eight‐ten cycle using the same oil. The percentage of DHA and EPA 7.67% and 0.65%, respectively, raw fish fillet and 0.87 and 0.20% found in fried fish fillet respectively, this shows the frying activity reduces the content of both DHA and EPA in fish fillet, and specially, a pronounced reduction was observed in DHA by frying practice. The other researchers did confirm as frying decreases EPA and DHA in all frying treatments independent of the fat used depends most probably on the fact that these fatty acids were not present in the frying fats (Gladyshev et al., 2007; Leung et al., 2018; Sampels et al., 2014).

The *n*‐6*/n*‐3 ratio is useful for determining fatty acid quality of food products. Lower ratio indicates that the food is very helpful in preventing coronary heart disease, whereas the higher ratio increases the risk (Beigi et al., 2014; Estuary et al., 2020). The ratio was 7.83 for fried fish muscle and 0.90 for raw fish muscle indicating increased in the fried sample. The ratio of polyunsaturated fatty acids to the saturated fatty acids is commonly used as another index. This index is very useful to reflect both effects of unsaturated mainly polyunsaturated and saturated fatty acids. Hence, a ratio of less than 0.45 is accounted as undesirable as the higher could probably increase the cholesterol in the blood (Estuary et al., 2020; Flores et al., 2018; Jayasena, Fernando, & Awanthika, 2018). In the current study, the ratio is 0.216 in the fried and 0.474 in the raw fish muscle. Therefore, the ratio is slightly below the desirable figure in the fried muscle.

Additionally, it is very important to note overall nutritional qualities of fat with the inclusion of monounsaturated fatty acids. In this case, IA, IT, and HH are crucial indicating the functional effects of fatty acids for human health. The high index of atherogenicity (IA) and thrombogenicity (IT) values is responsible for the formation of atheroma and stimulates aggregation of platelets in our cardiovascular system. Hence, the lower values of these indices are desirable as they are considered to prevent the cardiovascular disorders (Estuary et al., 2020; Karimian‐khosroshahi et al., 2016). In the current study, IA and IT were found to be higher in the fried fish muscle indicating a low nutritional quality of fried fish compared with the raw fish muscles. To this end, the HH is another indicator for the effect on cholesterol metabolism. The higher value of this index is considered to be desirable. Thus, in the current study the ratio is relatively similar in the fried (1.09) and raw (1.02) fish muscles (Estuary et al., 2020; Karimian‐khosroshahi et al., 2016).

Therefore, there implies that deep frying could result in reduction of the nutritional quality indices (NQI) related to fatty acid composition. The changes in reduction of the ratio are also similarly documented in other studies as deep frying could change the fatty acid compositions. However, the studies confirmed also the medium of frying(oil) could also change due to their contents of different fatty acids (Busra, Dagtekin, Misir, Kutlu, & Basturk, 2018; Djoussé, Petrone, & Michael Gaziano, 2015; Ganbi, 2011; Jayasena et al., 2018; Robert et al., 2014).

The level of *Tran's* fatty acid was 1.28% and 4.02% in raw and fried fish muscle tissue, respectively. Compared with the raw fish, there is a slight higher percentage in the fried fish muscle. There was significant effect on increasing the *Trans* fatty acid level (*p* < .001). This indicates that the unsaturated fatty acids could probably have the tendency to be converted to *Tran's* fatty acid due to frying practice. There are ample evidences showing the formation of *Tran's* fatty due to frying. For instance, *Tran's* isomers may be produced during processing of unsaturated oils (Rustan, 2005). Additionally, heating and frying causes geometrical isomerization leading *Tran's* fatty acid formation. During heating and frying, the geometrical isomerization of double bonds leads to the formation of *Tran's* fatty acids. During the reaction of oxygen and unsaturated fatty acid, hydroperoxides will form and a further radical reaction takes place due to temperature. Actually, common frying processes at about 170–180°C do not increase the *Trans* fatty acid content to a high extent. However, significant change of *Tran's* fatty acid during frying might happen due to the oil exchange between the frying good and the frying media. Deep‐fat frying at 180°C or above is one of the most common food processing methods used for preparing of human kind foods worldwide. However, a serial of complex reactions, such as oxidation, hydrolysis, and isomerization, takes place during the deep‐fat frying course and influencing the formation of *Tran's* fatty acid and other nutrients (Brühl, 2014; Hypophthalmus, 2011; Rahimabadi & Dad, 2012).

## 5 CONCLUSIONS AND RECOMMENDATION

The analysis of the current study shows that the dominant saturated fatty acids were palmitic, stearic, heptadecanoic, and tetradecenoic acids. Oleic, docosahexaenoic, eicosapentaenoic, and linoleic acid were the major unsaturated fatty acids. Fried fish muscles considerably contain higher percentages of saturated and *Tran's* fatty acids compared with the raw. To the opposite, the unsaturated fatty acids were lower in the fried fish muscles. The *n*‐6/*n*‐3 ratio, index of atherogenicity, and thrombogenicity in the fried fish muscle were in the undesirable nutritional quality values. The hypocholesterolemic/hypercholesterolemic ratio is relatively similar in the fried and raw fish muscles. This is an indication of frying fish muscle considerably alters the fatty acid compositions due to either the type of frying oil or deep frying by itself. Hence, it is recommended further analysis on the oils used for frying fish to identify which fatty acids are contributed to the fried muscle. Additionally, it is suggested that the effects of frying cycles on fatty acid composition are important to further identify the fatty acid‐related nutritional quality indices.

## CONFLICT OF INTEREST

The author(s) declare that there is no conflict of interest for authorship and/or publication of this article.

## AUTHOR CONTRIBUTIONS

DTD was a principal investigator, designed the study, took part in the sample collection process, laboratory experiment, entered and analyzed the data, and wrote the manuscript of the current study. FRA, MFM, GNK, and AKD oversaw the whole sample collection processes, helped in execution of laboratory experiments, data entry, and analysis, and helped for manuscript write up. All authors read and approved the final manuscript to be submitted.

## Data Availability

The datasets used and/or analyzed during the current study are available from the corresponding author on reasonable request.
